# Misconception of dementia-related disorders in Sub-Saharan Africa

**DOI:** 10.3389/fneur.2023.1148076

**Published:** 2023-04-17

**Authors:** Abdulyekeen T. Adebisi, Mufutau A. Salawu

**Affiliations:** ^1^School of Electronic and Electrical Engineering, Kyungpook National University, Daegu, Republic of Korea; ^2^Department of Physics, University of Ilorin, Ilorin, Nigeria

**Keywords:** Alzheimer's disease, awareness creation, dementia-related disorders, geriatric aging, Sub-Saharan Africa (SSA), witchcraft

## Introduction

Undoubtedly, the world has witnessed an unprecedented improvement in healthcare advancement and prosperity in the past few decades, and as a result, there has been an increase in the average life expectancy globally. Despite the progress, SSA still faces numerous health challenges such as high rates of infectious diseases, such as HIV/AIDS, malaria, and tuberculosis, limited access to healthcare services and medicines, inadequate health infrastructure, and a shortage of healthcare workers. Specifically, the aging population in the region is projected to grow rapidly (1, 2), which presents both challenges and opportunities. It is important for policymakers to address the needs of the aging population and plan for their well-being, such as access to healthcare, social security, and pension systems. At the same time, the aging population also represents a potential market for goods and services, and a source of experience and knowledge for the workforce. The increase in the aging population does come with associated challenges, such as the burden of age-related diseases such as dementia (3). The rise in the number of people living with dementia in the region is a major concern as it can strain the already limited healthcare resources and impact the quality of life for individuals and their families.

Unfortunately, traditional beliefs, inadequate awareness, and misconceptions about dementia and other age-related diseases have been contributing to poor management and understanding of these conditions in the Sub-Saharan Africa region (4–7). It is crucial to educate the public and healthcare providers about the true nature of dementia and other age-related diseases and to dispel common misconceptions. Therefore, this article emphasizes the need to address these misconceptions and educate people about the realities of dementia and related disorders in order to provide appropriate care and support to those affected.

## Dementia-related disorders

Dementia is a group of symptoms that affects memory, thinking, behavior, and the ability to perform everyday tasks (8, 9). There are many different types of dementia, including Alzheimer's disease, which is the most common cause of dementia (10). Other forms of dementia include vascular dementia, Lewy body dementia, and frontotemporal dementia (11, 12). All forms of dementia result in a progressive decline in cognitive function and can have a significant impact on a person's quality of life. Generally, the symptoms of different types of dementia can often overlap, making it difficult to diagnose the specific type without additional testing (13). Early symptoms of dementia can be subtle and may include mild memory loss, difficulty completing familiar tasks, and changes in mood and behavior. The general symptoms of dementia can include:

- Memory loss: This is a common symptom of dementia, especially in the early stages. People with dementia may have trouble remembering recent events or important information.
- Hallucination: This can include delusional experience or paranoia.
- Difficulty with language: This can include trouble in finding the right words, difficulty in understanding language, or problems with speaking or writing.
- Problems with visual and spatial abilities: This can include difficulty judging distances or interpreting visual information.
- Decreased reasoning and judgment: People with dementia may have trouble making decisions or solving problems.
- Impaired attention: They may have difficulty staying focused on a task or activity.
- Personality and behavior changes: This can include depression, anxiety, irritability, and mood swings.
- Loss of motivation: They may lose interest in hobbies or activities they once enjoyed.
- Inability to perform daily tasks: They may have difficulty with tasks such as cooking, cleaning, or managing finances.

It is important to note that dementia can affect different people in different ways, and symptoms may vary depending on the underlying cause of the condition. A thorough evaluation by a healthcare provider is necessary to determine the cause of any cognitive symptoms and to develop an appropriate treatment plan. These symptoms can often be mistaken for normal aging or stress (14), making it important for individuals to speak with a healthcare professional if they are concerned about their cognitive function.

The prevalence of dementia has been increasing due to the aging global population. It is estimated that 57 million people were living with dementia-related disorders in 2019, and with the growing number of older adults, this number is expected to rise in future (15, 16). As the global population ages, the number of people living with dementia is expected to increase dramatically. The estimate for 2050 is that there will be 152.8 million people with dementia, nearly three times the current number. Similarly, dementia has been one of the leading causes of death worldwide for several years (17), ranking as high as the third leading cause of death in some countries. The high death rate and increasing prevalence of dementia make it a major public health challenge that requires a coordinated global response. All the above highlight the urgent need for better understanding, prevention, and treatment of dementia.

The increasing prevalence of dementia has become a major public health concern, particularly in developed countries, where a larger proportion of the population is aging. As a result, there has been a significant increase in investment in research and development of healthcare strategies, including preventative measures, treatments, and therapies for dementia and related disorders. The goal is to improve the quality of life for people with dementia and to help reduce the overall burden of the disease on individuals, families, and society. In fact, the cost of caring for individuals with dementia is substantial and continues to rise as the number of cases increases. The estimated global cost of dementia in 2019 was around *$*818 billion US dollars, and this cost is expected to increase as the number of people with dementia grows in the coming years (18). The cost includes direct expenses such as medical care and indirect expenses such as lost income and productivity. The high cost of dementia highlights the need for effective treatments and preventative measures to reduce the economic and personal toll of the disease.

However, despite the increasing global burden of dementia, the situation in Sub-Saharan Africa (SSA) has received limited attention (19). The region faces many challenges in addressing the issue, including limited resources, lack of trained healthcare providers, and a lack of awareness and understanding of the disease. These challenges make it difficult to provide adequate care and support to individuals living with dementia and their families in the region. It is important for international organizations and governments to prioritize the issue and invest in developing effective strategies to improve the situation in SSA and other under-resourced regions. In many communities in Sub-Saharan Africa, there is a lack of understanding and awareness of dementia, and the symptoms are often misunderstood or attributed to other causes such as evil spirit possession (19). This can lead to a delay in diagnosis and treatment and result in stigma and discrimination toward individuals with the disease and their families. Additionally, the limited resources and weak healthcare systems in the region also contribute to the under-recognition and under-diagnosis of dementia. As a result, many people with dementia in SSA do not receive the care and support they need.

## Evil spirit possession

Spirit possession is a belief found in various cultures and religions around the world. It is typically characterized by a belief that a non-physical entity (20), such as a spirit, demon, or deity, has taken control of a person's body or mind, causing them to exhibit unusual or altered behaviors. The concept of evil spirit possession is often associated with certain cultural and religious beliefs (21, 22). In some cultures, it is believed that an individual can become possessed by a malevolent spirit or demon, which can cause a range of physical and psychological symptoms. The following are some common symptoms associated with evil spirit possession:

- Unusual behavior: A person who is possessed by an evil spirit may exhibit unusual or out-of-character behavior, such as speaking in tongues, exhibiting excessive violence, or engaging in self-harm.
- Personality changes: A person who is possessed may experience sudden and dramatic changes in their personality or mood, such as becoming withdrawn or aggressive.
- Loss of control: A person who is possessed may feel as though they are losing control over their thoughts, feelings, and actions.
- Physical symptoms: Possession can also cause physical symptoms such as convulsions, seizures, and paralysis.
- Unexplained illnesses: An individual who is possessed may experience unexplained illnesses or ailments that cannot be explained by medical science.
- Fear or aversion: Those around the possessed individual may feel an inexplicable sense of fear or aversion toward them.

It is important to note that many of these symptoms can also be caused by underlying mental health or medical conditions, and a professional evaluation is necessary to determine an accurate diagnosis and treatment plan. Additionally, belief in evil spirit possession is not universally accepted or recognized by all cultures and religions. While it is true that some of the symptoms of dementia, such as confusion, agitation, and hallucinations, may bear similarities to the symptoms associated with demonic or evil spirit possession in certain cultural contexts (23), it is important to note that these are two distinct phenomena with different causes. Dementia is a well-researched and well-understood medical condition, and it can be diagnosed through medical examinations and tests. On the contrary, the belief in demonic possession is typically based on religious or cultural beliefs and is not recognized as a medical condition in most contexts. Therefore, it is important to approach any individual who may be exhibiting symptoms of confusion, agitation, or hallucinations with compassion and an open mind but to seek medical attention for a proper diagnosis and treatment plan.

## Witchcraft and bewitchment

Historically, witchcraft has been associated with the use of supernatural powers or magic to achieve certain outcomes or to influence events (24). In some cultures, witchcraft is seen as a form of religious or spiritual practice, while in others, it is viewed as a form of occultism or sorcery. However, it is important to note that the idea of witchcraft as “supernatural interference in the natural community lifestyle and behavior” is a subjective interpretation and may not be accurate or applicable to all cultures and beliefs (25). Different societies and communities have their unique perspectives on witchcraft, and these can vary widely depending on factors such as geography, religion, and cultural norms. Confessing to witchcraft in the SSA region may involve a range of psychological and emotional symptoms (26):

- Fear: Confessing about witchcraft may be accompanied by feelings of fear, anxiety, and uncertainty, especially if the individual is facing persecution or punishment for their beliefs.
- Guilt: Individuals who confess to witchcraft may experience feelings of guilt or shame, particularly if their beliefs are contrary to their cultural or religious norms.
- Hallucinations: In some cases, individuals who confess to witchcraft may report experiencing hallucinations or other sensory experiences that they believe are linked to their beliefs.
- Paranoia: Confessing to witchcraft may also lead to feelings of paranoia or suspicion, especially if the individual believes that they are being persecuted or targeted by others.

It is important to recognize that witchcraft beliefs and practices are complex and multifaceted and that a supposed confession to witchcraft is quite elusive especially when it has to be proven scientifically. In SSA, older women are one of the most vulnerable members of the society at risk of being accused of witchcraft (27). As it could be noted, the psychological and emotional symptoms associated to witchcraft confession overlap with the symptoms of dementia disorders (6). Therefore, without clinical validation, it becomes so easy to misconceive dementia symptoms in older adults with witchcraft.

## Dementia-related disorders and traditional beliefs in SSA

Belief in witchcraft and evil spiritual possession is prevalent in some societies and has a long history in African traditional beliefs (28, 29). However, it is important to note that African worldviews are diverse and complex, and this belief is not universally held across all African cultures. Some African societies have different beliefs and practices related to spirituality and supernatural forces. However, education and modernization can impact people's beliefs, but the influence of traditional beliefs and cultural practices can persist even among those with higher levels of education. Despite efforts to promote education and modernization in many countries in sub-Saharan Africa (SSA), the extremism of belief in witchcraft and evil spiritual possession continues to persist in some communities in the region. Such extremism is often deeply rooted in cultural and historical traditions and is often sustained by personal experiences and community practices. As a result, it remains an important aspect of some people's lives and continues to play a significant role in shaping their beliefs and practices.

The continuous popularity of harmful belief in witchcraft and evil spiritual possession in some communities in sub-Saharan Africa is however due to a lack of access to comprehensive geriatric care and gerontological education. This often results in misunderstandings about the symptoms of age-related health conditions, including dementia. Consequently, this usually led to a belief that the symptoms of dementia are caused by witchcraft or evil spirits possession. On the contrary, some religious leaders and traditional healers in SSA often exploit people's beliefs for financial gain. In some instances, they claim to induce witchcraft confession and thereby subject the victims (who are usually older adults) to public assaults. In some other instances, religious leaders and traditional exploitation take the form of claiming the ability to deliver spiritually possessed individuals from spiritual afflictions. This can result in individuals with dementia and their families seeking out these religious leaders and traditional healers for help rather than seeking medical treatment from trained healthcare professionals. Hence, such reinforces perpetuates the belief in witchcraft and evil possession and further marginalizes people who are suffering from age-related health conditions, including dementia.

Meanwhile, as dementia progresses, it is not uncommon for a person living with dementia to experience difficulties in performing daily activities and may also experience changes in social behavior and emotional well-being. It is important to understand that these symptoms are a result of a medical condition and may not be necessarily due to witchcraft or evil possession and that individuals living with dementia deserve compassion, support, and access to appropriate care. On the contrary, in some communities in SSA, where harmful belief in witchcraft and evil spiritual possession is prevalent, these symptoms are misconstrued. In these communities, individuals with symptoms of dementia may be misperceived as being possessed by evil spirits and may be subjected to harmful cultural practices, stigmatization, public abuse, or other punishments. It is therefore important to address these harmful beliefs and practices through education and awareness-raising campaigns. At the same time, it is important to recognize the value of religious leaders and traditional healing practices in some communities, while ensuring that they do not take the place of evidence-based medical care. Collaboration between traditional healers and healthcare professionals can lead to more effective and culturally sensitive care for individuals with dementia in SSA, especially with the availability of research-based validation of evil spirit possession and witchcraft.

## Government policy

The lack of government policies and actions to protect the geriatric population from human rights violations, particularly those with dementia, is a major concern in SSA and Africa (30–32). This is because age-related disease symptoms are quite similar to those symptoms perceived to be caused by evil spirit possession and bewitchment which older adults are commonly accused of. Therefore, the older population in these regions is at a higher risk of abuse, neglect, and mistreatment, and the absence of policies to protect them only exacerbates their vulnerability. Unfortunately, governments and policymakers in SSA have not been paying adequate attention to the aging population despite the call of the UN in this regard (33). It is then essential for SSA governments, policymakers, and relevant stakeholders to prioritize the rights and well-being of older adults with dementia by developing and implementing policies and programs that provide them with the care and support they need. This includes measures to prevent abuse and neglect, promote their autonomy, and improve their access to healthcare, social services, and legal protections.

## Recommendations

Education is crucial in promoting awareness and understanding of dementia-related disorders and the rights of older adults. The inclusion of gerontology and geriatric education in school curricula will help to break down stereotypes and misconceptions, while also equipping the next generation with the knowledge to provide better care and support to older adults. Additionally, education can play a role in reducing abuse and human rights violations against older adults living with dementia by raising awareness and promoting empathy.

Similarly, it is important for governments to take steps to protect vulnerable populations, including older adults and those living with dementia-related disorders, from exploitation and abuse by religious organizations or other entities. Establishing policies that restrict the use of spiritual warfare or deliverance for financial gain and enforcing penalties for those who violate these policies can help reduce the harm caused by such practices. Additionally, education and awareness campaigns can help counter these harmful beliefs and promote evidence-based approaches to care for older adults and those living with dementia.

In addition, having well-equipped geriatric homes and institutions is important in providing safe and appropriate care for older adults and those living with dementia-related disorders. This can help to reduce the risk of abuse and neglect and provide a supportive environment for individuals who are unable to live independently. Providing adequate resources for geriatric care, including funding for research and training programs, will also help to attract and retain medical professionals with expertise in geriatric medicine. This will help to improve the quality of care for older adults and those with dementia and to reduce the incidence of human rights violations and abuse in these populations.

Further, it is essential to encourage and support African researchers in their efforts to study and address dementia-related disorders. By providing the necessary training, equipment, and resources, researchers will be better equipped to carry out high-quality research and make significant contributions to the field. Collaboration between African researchers and international organizations working in the field of dementia research can also help to advance knowledge and develop effective solutions to address the challenges posed by dementia-related disorders in the SSA region and Africa as a whole. Additionally, partnerships between governmental and non-governmental organizations can help to create a supportive environment for dementia-related research, encouraging further investment and progress in the field.

## Conclusion

Harmful traditional beliefs in witchcraft and spiritual possession are still prevalent in the SSA region and can lead to stigma for older adults with dementia. It is important to raise awareness about dementia and its symptoms through sensitization programs to help dispel misconceptions of its symptoms with evil spirit possession and bewitchment. It is also important for governments in the SSA region to prioritize establishing geriatric education policies to improve understanding of dementia and reduce older adult stigmatization in society.

## Author contributions

AA: conceptualization, study design, data collection and analysis, and drafting and revision of the manuscript. MAS: conceptualization and drafting and revision of the manuscript. Both authors contributed to the article and approved the submitted version.

## References

[1] Aboderin IA Beard JR. Older people's health in sub-Saharan Africa. Lancet. (2015) 385:e9e11. 10.1016/S0140-6736(14)61602-0 25468150

[2] Bigna JJ Noubiap JJ. The rising burden of non-communicable diseases in sub-Saharan Africa. Lancet Global Health. (2019) 7:e1295–6. 10.1016/S2214-109X(19)30370-5 31537347

[3] Christensen K Doblhammer G Rau R Vaupel JW. Ageing populations: the challenges ahead. lancet. (2009) 374:1196–208. 10.1016/S0140-6736(09)61460-4 19801098 PMC2810516

[4] Spittel S Kraus E Maier A. Dementia awareness challenges in Sub-Saharan Africa: a cross-sectional survey conducted among school students in Ghana. Am J Alzheimers Dis Other Demen. (2021) 36:15333175211055315. 10.1177/15333175211055315 34985361 PMC10581119

[5] Spittel S Maier A Kraus E. Awareness challenges of mental health disorder and dementia facing stigmatisation and discrimination: a systematic literature review from Sub-Sahara Africa. J Glob Health. (2019) 9:020419. 10.7189/jogh.09.020419 31656607 PMC6790232

[6] Brooke J Ojo O. Contemporary views on dementia as witchcraft in sub-Saharan Africa: A systematic literature review. J Clin Nurs. (2020) 29:20–30. 10.1111/jocn.15066 31531993

[7] Khonje V Milligan C Yako Y Mabelane M Borochowitz K De Jager C. Knowledge, attitudes and beliefs about dementia in an urban Xhosa-speaking community in South Africa. Adv Alzheimers Dis. (2015) 4:21. 10.4236/aad.2015.42004

[8] Filley C. Alzheimer's disease: it's irreversible but not untreatable. Geriatrics. (1995) 50:18–23. 7601358

[9] Sachdev PS Blacker D Blazer DG Ganguli M Jeste DV Paulsen JS. Classifying neurocognitive disorders: the DSM-5 approach. Nat Rev Neurol. (2014) 10:634–42. 10.1038/nrneurol.2014.181 25266297

[10] Liang CS Li DJ Yang FC Tseng PT Carvalho AF Stubbs B. Mortality rates in Alzheimer's disease and non-Alzheimer's dementias: a systematic review and meta-analysis. Lancet Healthy Longevity. (2021) 2:e479–88. 10.1016/S2666-7568(21)00140-9 36097997

[11] Fereshtehnejad SM Garcia-Ptacek S Religa D Holmer J Buhlin K Eriksdotter M. Dental care utilization in patients with different types of dementia: a longitudinal nationwide study of 58,037 individuals. Alzheimers Dementia. (2018) 14:10–19. 10.1016/j.jalz.2017.05.004 28692821

[12] Galende AV Ortiz ME Velasco SL Luque ML de Miguel CLdS Jurczynska CP. Report by the Spanish Foundation of the Brain on the social impact of Alzheimer disease and other types of dementia. Neurología. (2021) 36:39–49. 10.1016/j.nrleng.2017.10.004 29249303

[13] Folstein MF. Differential diagnosis of dementia: the clinical process. Psychiatr Clin North Am. (1997) 20:45–57. 10.1016/S0193-953X(05)70392-0 9139295

[14] Gauthier S Reisberg B Zaudig M Petersen RC Ritchie K Broich K. Mild cognitive impairment. Lancet. (2006) 367:1262–70. 10.1016/S0140-6736(06)68542-5 16631882

[15] Nations U. World Population Ageing 2019: Highlights. New York, NY: United Nations (2019).

[16] Nichols E Steinmetz JD Vollset SE Fukutaki K Chalek J Abd-Allah F. Estimation of the global prevalence of dementia in 2019 and forecasted prevalence in 2050: an analysis for the Global Burden of Disease Study (2019). The Lancet Public Health. (2022) 7:e105e125. 10.1002/alz.051496 34998485 PMC8810394

[17] Nichols E Szoeke CE Vollset SE Abbasi N Abd-Allah F Abdela J. Global, regional, and national burden of Alzheimer's disease and other dementias, 1990-2016: a systematic analysis for the Global Burden of Disease Study 2016. Lancet Neurol. (2019) 18:88–106. 10.1016/S1474-4422(18)30403-4 30497964 PMC6291454

[18] Rodgers PA. Design research for change: caring for people living with dementia: introduction. In: Design for People Living with Dementia. London: Routledge (2022). p. 1–12.

[19] Dotchin C Paddick SM Longdon A Kisoli A Gray W Dewhurst F. A comparison of caregiver burden in older persons and persons with Parkinson's disease or dementia in sub-Saharan Africa. Int Psychogeriatr. (2014) 26:687–92. 10.1017/S104161021300255X 24507385

[20] Rashed MA. More things in heaven and earth: spirit possession, mental disorder, and intentionality. J Med Human. (2020) 41:363–78. 10.1007/s10912-018-9519-z 30027384 PMC7343734

[21] Pietkiewicz IJ Kłosińska U Tomalski R van der Hart O. Beyond dissociative disorders: a qualitative study of Polish catholic women reporting demonic possession. Eur J Trauma Dissociat. (2021) 5:100204. 10.1016/j.ejtd.2021.100204

[22] Ventriglio A Bonfitto I Ricci F Cuoco F Bhavsar V. Delusion, possession and religion. Nordic J Psychiatry. (2018) 72:S13–5. 10.1080/08039488.2018.1525639 30489188

[23] Fotuhi M Hachinski V Whitehouse PJ. Changing perspectives regarding late-life dementia. Nat Rev Neurol. (2009) 5:649–58. 10.1038/nrneurol.2009.175 19918254

[24] Douglas M. Witchcraft Confessions and Accusations. London: Routledge (2013).

[25] Jayeola-Omoyeni M Oyetade EM Omoyeni J. Witchcraft in the 20th and 21st Centuries in Nigeria: an analysis. Eur Sci J. (2015) 11:28. Available online at: https://eujournal.org/index.php/esj/article/view/6396

[26] Adinkrah M. Child witch hunts in contemporary Ghana. Child Abuse Neglect. (2011) 35:741–52. 10.1016/j.chiabu.2011.05.011 21943497

[27] Bastian ML. The Daughter she will Eat Agousie in the World of the Spirits Witchcraft Confessions in Missionised Onitsha, Nigeria. Africa. (2002) 72:84–111. 10.3366/afr.2002.72.1.84 21038711

[28] Asamoah-Gyadu JK. Witchcraft accusations and Christianity in Africa. Int Bull Missionary Res. (2015) 39:23–7. 10.1177/239693931503900107

[29] Mkhonto F Hanssen I. When people with dementia are perceived as witches. Consequences for patients and nurse education in South Africa. J Clin Nurs. (2018) 27:e169–76. 10.1111/jocn.13909 28557051

[30] Douglass R. The aging of Africa: Challenges to African development. Afr J Food Agric , *Nutr Dev*. (2015) 16:1–15.

[31] Tanyi PL André P Mbah P. Care of the elderly in Nigeria: Implications for policy. Cogent Soc Sci. (2018) 4:1555201. 10.1080/23311886.2018.1555201

[32] Kpessa-Whyte M Tsekpo K. Lived experiences of the elderly in Ghana: Analysis of ageing policies and options for reform. J Cross Cultural Gerontol. (2020) 35:341–52. 10.1007/s10823-020-09401-z 32440805

[33] Aboderin I. Understanding and advancing the health of older populations in sub-Saharan Africa: policy perspectives and evidence needs. Public Health Rev. (2010) 32:357–76. 10.1007/BF03391607

